# Conservation of the Patchily Distributed and Declining Purple-Crowned Fairy-Wren (*Malurus coronatus coronatus*) across a Vast Landscape: The Need for a Collaborative Landscape-Scale Approach

**DOI:** 10.1371/journal.pone.0064942

**Published:** 2013-05-29

**Authors:** Anja Skroblin, Sarah Legge

**Affiliations:** 1 Research School of Biology, Australian National University, Canberra, Australia; 2 Australian Wildlife Conservancy, Subiaco, Perth, Australia; 3 School for Environmental Research and North Australian Biodiversity Hub, Charles Darwin University, Casuarina, Northern Territory, Australia; Università degli Studi di Napoli Federico II via Università, Italy

## Abstract

Conservation of species that are patchily distributed must consider processes that influence both the occurrence of individuals within patches, and the persistence of populations across multiple habitat patches within the landscape. Here we present a rare regional assessment of the population size and distribution of a patchily distributed, threatened species, the purple-crowned fairy-wren (*Malurus coronatus coronatus*), across a vast landscape. We used data from aerial vegetation mapping of waterways, with on-ground bird surveys to predict the occurrence of suitable habitat for *M. c. coronatus* across 14 catchments in the Kimberley region of Western Australia. Suitable habitat was extremely limited (305 km of riparian vegetation) and fragmented (342 patches) along the 2700 km of waterway surveyed within catchments where the species occurs. Populations were predicted to be large on the Fitzroy, Durack and Drysdale catchments, and small on the Isdell and northern Pentecost catchments, and a total population of 2834 to 4878 individuals could be supported. The sub-populations spanned numerous patches of habitat across multiple properties of varying tenure. Therefore, a landscape-scale approach to conservation management, across multiple tenures, is critical to safe-guard connectivity within populations. The greatest benefit may be achieved by a combination of broad-scale actions to reduce the impact of ubiquitous threatening processes, and fine-scale targeted effort in areas where populations are most vulnerable. Controlling access of stock to waterways and management of fire are most important to conserve suitable habitat. Such a landscape-scale approach to conservation may be of benefit to other patchily distributed species.

## Introduction

Conservation of species that are distributed across small and isolated habitat patches presents specific challenges for managers. The persistence of sub-divided populations is influenced by factors operating at multiple scales [1], [2]. This includes variation in fine-scale factors such as the quality of habitat within patches [3], [4], to landscape-scale factors such as spatial arrangement and abundance of habitat [5], the condition of the matrix [6], [7], and connectivity between isolated populations [8]–[10]. The extent to which populations are affected by such processes is influenced by the life-history of species and the landscape context [11].

In order to allocate conservation effort effectively at the level of sub-populations, knowledge is required of factors influencing the fine-scale distribution of populations [12], the spatial and temporal variation in threatening processes [13], [14], and the availability of suitable habitat at a landscape-scale [15], [16]. Here we present a study which predicts patch-scale occurrence of the endangered purple-crowned fairy-wren (*Malurus coronatus coronatus*) across a vast landscape, to guide management actions for sub-populations that are distributed across widely-dispersed habitat patches.

The purple-crowned fairy-wren (*Malurus coronatus*) is a riparian habitat specialist that is restricted to small, widely-dispersed patches of lush vegetation that grow along the waterways of northern Australia. The species is pressured by the on-going degradation and loss of riparian habitat caused by grazing and trampling by introduced herbivores [17]–[19], weed incursion, and frequent intense fires [20]–[22]. The Vulnerable [23] western sub-species (*Malurus coronatus coronatus*) has continued to decrease in distribution and abundance in response to these processes [17], [24], [25].

Until recently, the distribution of the western purple-crowned fairy-wren was poorly described due to the remoteness of the region in which it occurs; available records were mostly limited to a very small number of well visited locations [25]. An extensive survey for the species across the 14 catchments in the Kimberley section of its range has addressed that knowledge gap: purple-crowned fairy-wrens are distributed across a large number of small habitat patches on widely dispersed waterways within five catchments [25]. Birds on each of these five catchments are genetically divergent (Skroblin unpublished data), indicating that sub-populations are poorly connected and dispersal between catchments is restricted.

Given their scattered distribution and population structure [25], the species required a region-wide assessment of the extent and location of populations to evaluate the most appropriate approach to ensure the persistence of population processes and key sub-populations. This study describes the fine-scale distribution of suitable habitat to inform such an approach within the Kimberley region of Western Australia. We use bird survey and vegetation assessment data to develop a predictive model for assessing the suitability of riparian vegetation for the purple-crowned fairy-wren from aerial surveys. Using this tool, we then 1) estimate the extent of suitable habitat in the region, 2) predict the location and size of sub-populations, and 3) summarise the availability of habitat with respect to land tenure (and thus potential variation in threats). This information enables us to identify the most effective management model for their long-term conservation, evaluate the risk of decline of each subpopulation and assess whether any currently ‘unoccupied’ waterways contain sufficient habitat for purple-crowned fairy-wrens.

## Materials and Methods

### Ethics and permit statement

The field study protocols were approved by the Australian National University Ethics Committee (F.BTZ. 07.07), and carried out under permits from the Australian Bird and Bat Banding Scheme (2770), and Western Australian State Government (WA: BB002411). We thank landholders for their permission to access the properties that were surveyed.

### Survey design

The study was conducted in the Kimberley region of Western Australia during the dry seasons of 2007 (May–Oct), 2008 (May–Oct) and 2009 (July). We sought to map the distribution of a large number of highly dispersed habitat patches in a region with very limited road access. Ground surveys were therefore not possible. Standard remote sensing techniques were also inadequate because we needed to classify understorey vegetation beneath a canopy [26], [27]. Consequently, aerial survey using an R44 helicopter and a handheld GPS (Garmin GPSmap 60, Schaffhausen, Switzerland) was found to be the most appropriate way to map and describe the riparian vegetation.

In order to generate accurate estimates for habitat extent and population sizes, the aerial surveys were geographically extensive. They included all sections of waterways within the region where the purple-crowned fairy-wren has been recorded (The Atlas of Australian Birds, September 1998–July 2007, Birds Australia, Melbourne; [17], [25]), and all sections of waterway where dense riparian vegetation could be identified from low-resolution satellite images (Google Earth). Specifically, surveys traversed 1) all five catchments where the species currently occurs (Fitzroy, Durack, Isdell, Drysdale and Pentecost), 2) a section of the Ord catchment from which the species recently disappeared, and 3) sections of eight additional catchments (Sale-Berkelman, Forrest, Berkeley, King George, Calder, Charnley and Carson) that may contain suitable habitat, but on which purple-crowned fairy-wrens have never been recorded [25]. Surveys did not include the Victoria River section of the species distribution where habitat has been previously surveyed [24], or rivers in the north-western Kimberley (in the Prince Regent Nature Reserve and on the Mitchell Plateau) that have often been visited by biologists and purple-crowned fairy-wrens have never been recorded. In total, 47 sections of waterway were surveyed (Table S1), and the surveys traversed 37 properties of varying tenure.

### Vegetation mapping

In the study region, riparian vegetation grows as narrow belts along rivers banks, thereby causing a linear arrangement of territories of the purple-crowned fairy-wren, which strictly depend on this vegetation [28], [29]. Territory size is best measured as the length of waterway held by a territorial group [28], and the length of suitable habitat along a waterway determines the number of territories that can be supported [17]. Based on this knowledge we used riparian habitat patch length as our metric of habitat extent.

### Vegetation attributes

Previous studies have identified the key vegetation characteristics that are a pre-requisite for occupancy by purple-crowned fairy-wrens. These are a dense mid-storey (of *Pandanus* and/or freshwater mangrove, or river grass), which is important for nesting and shelter, and a high canopy which acts as a temporary refuge during the flooding events that often occur during the summer monsoons [17], [19], [30]. We therefore mapped patches of vegetation that contained both canopy and understorey structure.

We selected a simple set of mid-storey and canopy attributes that could be reliably scored from aerial surveys. These were: the percentage of bank covered with either: 1) *Pandanus*, 2) tall river grasses (such as *Chionachne cyanthopoda*), and 3) shrubs; plus 4) the canopy cover across a patch; and 5) the height of canopy in relation to flood height. To enable surveys to be conducted rapidly, we recorded predictors as categorical values (Table 1). The location and extent of patches were recorded using a hand-held GPS, and a geographic information system (ArcGIS V9.2, ESRI) was used to determine the length of habitat patches, the number of patches, the total extent of riparian vegetation for each catchment, and to produce maps of vegetation configuration [31], [32].

**Table 1:** Vegetation attributes of patches recorded during aerial vegetation mapping.

Patch attribute	Median (range)	Description
Canopy height	5 (1–5)	1) Below flood level, 3) above flood level but <10 m, 5) above flood level and >10 m
Canopy cover	5 (1–5)	Continuity of over-storey: 1) <25%, 3) 25–75%, 5) >75%
Shrub cover	5 (1–5)	Mid-storey other than *Pandanus* and river grass: 1) <5%, 3) 5–50%, 5) >50% bank covered
*Pandanus* cover	2 (1–5)	Bank covered with *Pandanus*: 1) Absent, 2) <25%, 3) 25–50%, 4) 50–75%, 5) >75%
River grass cover	1 (1–5)	River grass cover: 1) none, 3) <50% river grass cover, 5) >50%

### Bird surveys

To develop the predictive habitat suitability model based on aerial vegetation assessments, we conducted on-ground surveys for the presence of purple-crowned fairy-wrens within vegetation patches. Surveys were conducted within a subset (113) of the aerially mapped patches on five catchments where the species currently occurs [25]. We did not include sites from outside the current distribution of the species, as absence from these areas may be influenced by limits to colonisation rather than the suitability of habitat [33].

**Figure 1:**
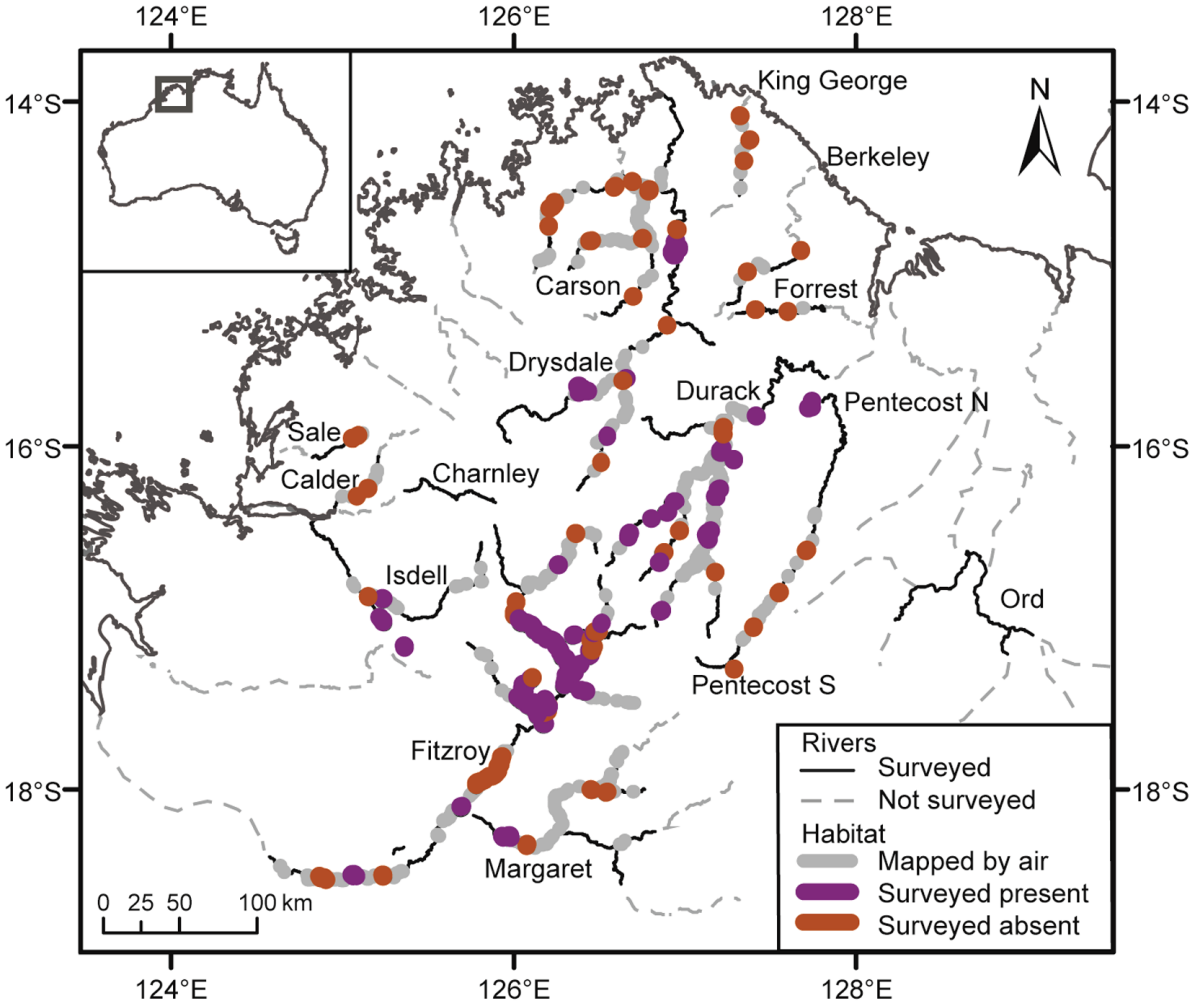
Riparian vegetation mapped during helicopter surveys of waterways in the Kimberley region of Western Australia. All patches that were mapped contained both canopy and mid-storey structure. Waterways that were surveyed are described in Table S1. These waterways were where sightings had previously been reported, and/or where riparian vegetation was discernable on low-resolution satellite imagery (Google Earth). Not all waterways that were surveyed contained potential habitat. On-ground bird surveys were conducted in the patches that are indicated in purple and orange. Only presence/absence data from catchments where the species has been confirmed to occur (Fitzroy, Durack, Isdell, Drysdale and Pentecost) were used to generate the predictive model of habitat suitability.

We surveyed for purple-crowned fairy-wrens in a minimum of three patches of vegetation on each section of river that was mapped by air, giving preference to patches that were long enough to contain territories (>300 m). Either the entire patch (if<1 km) or a minimum of 1 km of riparian vegetation was surveyed in each instance. In total, bird surveys were conducted within 79 patches on the Fitzroy, five patches on the Isdell, 12 patches on the Drysdale, three patches on the northern Pentecost, and 19 patches on the Durack catchments (Figure 1). Surveys were conducted following the reliable detection method of Skroblin and Legge [25]. Briefly, we walked within or along the edge of riparian vegetation and broadcast *M. c. coronatus* territorial calls to assist in detection of this highly territorial species [34].

### Statistical data analysis

The data from the patches that were surveyed on-ground for the presence of purple-crowned fairy-wrens were used to develop a logistic model to predict the suitability of mapped riparian vegetation. When developing this model, river grass cover was not included as an explanatory variable because it was only encountered at one of the patches that were surveyed on-ground, and only detected at five patches during aerial mapping. Modelling of habitat suitability was therefore limited to areas containing *Pandanus* or shrub dominated habitat. Prior to modelling, correlations between vegetation attributes were computed and examined for multicollinearity. Analyses were conducted in GenStat 11.1 (VSN International).

We used generalized linear modelling (GLM) with binomial error distribution and a logit link function [35] to evaluate the fit of twelve combinations of the patch vegetation attributes in explaining occurrence of the purple-crowned fairy-wren. These combinations all contained a mid-storey parameter and a canopy parameter, because both structures are known to be integral components of suitable habitat. Our modelling approach used the multi-modal inference framework [36], and employed the Akaike Information Criterion adjusted for small sample size (AIC_c_). Firstly, Akaike weights [37] were calculated for each candidate model relative to the likelihood of a model. An Akaike weight (ω*_i_*), is the weight of evidence in favour of a candidate model (*i*), being the best approximating model in the set of models available. We then obtained a 90% confidence set of plausible candidate models by summing Akaike weights of models (from smallest to largest) until the sum was ≥0.9. A weighted model-averaging approach was then employed to calculate the summed Akaike weights for each predictor variable and also the averaged partial regression coefficients from the models within the 90% confidence set [36]. For a secondary measure of model rankings, Adjusted R^2^ was also calculated [38]. Catchment was initially included as a random term to account for spatial structure of sampling but did not improve the fit of models to the data and was subsequently excluded.

The final model, containing the model-averaged partial regression coefficients, was fitted to the vegetation attributes of every patch mapped during aerial surveys. We performed inverse logistic transformation of the linear predictor to calculate habitat suitability as values between zero and one [31]. To delineate riparian vegetation into potentially *suitable* and *unfavourable* habitat, we identified the minimum predicted habitat suitability score at which purple-crowned fairy-wrens were found to be present during bird surveys. This threshold was used to identify which patches mapped during aerial surveys contained *suitable* habitat. Only patches that were classified as potentially *suitable* were included in summaries of habitat distribution across land tenure types and in calculations of population estimates, below.

### Distribution of suitable habitat across land tenure types

We summarized the availability of habitat with respect to land tenure (in 2013) by assigning habitat to categories based on five land tenures: 1) pastoral (pastoral land, including indigenous managed); 2) vacant Crown Land; 3) conservation (National Parks and Conservation Parks); 4) private conservation (Australian Wildlife Conservancy land with a pastoral history that is now managed for conservation); and 5) indigenous (indigenous land reserves, excluding indigenous pastoral). Where habitat patches were on waterways separating lands of differing tenure, the tenure of highest *theoretical* impact was assigned, i.e. habitat between conservation and pastoral land was assigned as pastoral.

### Population estimates

We estimated the number of territories and absolute population size that each catchment could potentially support, by combining information on demographic data [25], [28] with our map of suitable habitat. Because estimation of population size is complicated by variation in the number of birds within a territorial group and variation in the length of territories, we estimated upper and lower population estimates for each catchment to account for this variation. We calculated: 1) upper and lower 95% confidence intervals for the mean number of birds per territory (2.8 and 3.2) from group size data for 167 purple-crowned fairy-wren territories surveyed in the Kimberley region [25], 2) upper and lower estimates of mean number of territories per kilometre (3.34 and 5) using Rowley and Russell's [28] estimate of territories being between 200 to 300 m in length in the Kimberley region, and 3) the resulting upper and lower mean number of birds per kilometre of habitat (9.3 and 16). Absolute population size (*N*) was calculated as the product of the mean number of birds per kilometre of suitable habitat and the length of suitable habitat: *N* = average birds per km×length suitable habitat

## Results

### Survey findings

We surveyed approximately 4000 km of waterway within the Kimberley region, of which 490 km contained vegetation that included a canopy and mid-storey structure (Table 2). The highest extents of riparian vegetation were documented on rivers where the species occurs: the Fitzroy (241 km), Durack (98 km), and Drysdale (47 km) Rivers. Relatively little riparian vegetation was documented on catchments where the species does not occur, with the exception of the Carson catchment (61 km) (Table 2).

**Table 2:** Summary of the extent of riparian vegetation mapped during aerial surveys of 14 catchments within the Kimberley region.

Catchment	PCFW	Survey distance (km)	Riparian vegetation (km)	Number of patches
Fitzroy	Y	1316	241	207
Isdell	Y	236	12	23
Drysdale	Y	566	47	60
Durack	Y	641	98	113
Pentecost - north	Y	25	3	5
Subtotal		2784	401	408
Ord	N	367	0	0
Forrest	N	88	2	5
Berkeley	N	87	1.6	5
King George	N	63	7.2	14
Sale	N	41	2.6	4
Calder	N	141	2.7	10
Charnley	N	78	0	0
Pentecost - south	N	174	11	21
Carson	N	252	61	66
Subtotal		1213	88	125
Total		3997	490	533

Riparian vegetation that was mapped contained both canopy and mid-storey structure.

### Model of habitat suitability

Correlations between vegetation attributes were all less than r = 0.25 (Table 3) and thus multicollinearity between variables was low. The best approximating multivariate model of habitat suitability contained the predictors of shrub cover, *Pandanus* cover and canopy height (GLM, df = 3, 112, deviance ratio = 5.11, P = 0.002). This model had the lowest AIC_c_ value (Table 4); however the Akaike weight of 0.28 for this model suggests substantial model selection uncertainty. Moreover, support for the second model was also strong with a 0.047 difference in Akaike weights between the two (Table 4). Eight models were included in the 90% confidence set of plausible candidate models (Table 4), so the uncertainty of model 1 being the best model was considerable. Hence it was appropriate to undertake model averaging within the 90% confidence set of models to develop a predictive model of habitat suitability (Table 5).

**Table 3:** Correlations between patch attributes for catchments within the current range of the purple-crowned fairy-wren.

	Canopy cover	Canopy height	Shrub cover
Canopy height	0.151		
Shrub cover	0.118	0.229*	
*Pandanus* cover	0.117	0.192*	0.077

* Significance level of P<0.05. *N* = 113.

**Table 4:** Results of model selection using a multi-model inference framework for habitat suitability of riparian vegetation patches for the purple-crowned fairy-wren.

Rank	Explanatory variables	*K*	AIC_c_	Δ*_i_*	*L*(*g_i_*|*x*)	ω*_i_*	*AdjR^2^*
1	S+P+CH	4	116.62	0.00	1	0.280	10.44
2	S+P	3	116.99	0.37	0.83	0.233	9.31
3	S+P+CC+CH	5	118.56	1.94	0.38	0.106	9.66
4	S+CH	3	118.58	1.96	0.37	0.105	8.00
5	S+P+CC	4	118.97	2.35	0.31	0.087	8.49
6	P+CC	3	120.01	3.39	0.18	0.051	6.83
7	S	2	120.55	3.93	0.14	0.039	5.6
8	S+CC+CH	4	120.57	3.95	0.14	0.039	7.16
9	P+CC+CH	4	121.99	5.37	0.07	0.019	5.99
10	P	2	122.12	5.5	0.06	0.018	4.32
11	S+CC	3	122.52	5.9	0.05	0.015	4.76
12	P+CC	3	124.10	7.48	0.02	0.007	3.47

All evaluated models are shown, those above the line were included in the 90% confidence set of models. The table shows the number of terms in the model (*K*), Akaike Information Criteria adjusted for small sample size (AIC_c_), AIC_c_ differences (Δ*_i_*), the likelihood of model *i* given the data (*L*(*g_i_*|*x*)), Akaike weights (ω*_i_*), and Adjusted R-square (AdjR^2^). Explanatory variables: S = shrubs; P = *Pandanus*; CH = canopy height; CC = canopy cover.

**Table 5:** Model averaged coefficients, standard errors and weighting for each variable included in the 90% confidence set of models.

Explanatory terms	Coefficient	SE	Weight
Constant	−11.561	17.95	
Canopy cover	−0.014	0.08	0.246
Canopy height	2.068	3.51	0.619
Shrub cover	0.381	0.22	0.945
*Pandanus* cover	0.442	0.15	0.805

The final model, which predicts habitat suitability scores between zero and one, contained the model-averaged partial regression coefficients (Table 5):
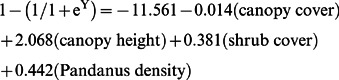
Our modelling approach indicated that a dense mid-storey of shrubs and *Pandanus*, and high canopy and continuity of canopy cover, when measured during helicopter survey, were appropriate predictors of purple-crowned fairy-wren occurrence (assessed in ground surveys) along the rivers surveyed in the Kimberley region (Table 4). This model however would be inappropriate to assess habitat suitability in areas where purple-crowned fairy-wren habitat is characterized by an understorey of river grass, i.e. lower Fitzroy River or Victoria River [19]. The most highly weighted and thus important predictors of occurrence in our model were shrub cover, *Pandanus* cover and canopy height, while canopy cover was less important (Table 5). The slightly negative coefficient of canopy cover (Table 5) may indicate that trees that are tall enough to provide refuge from flooding grow at low density along river verges. As 98% of fairy-wrens were detected in patches with suitability >0.5, we identified this as a threshold to purple-crowned fairy-wren occurrence. Thus we consider only patches with >0.5 as *suitable* habitat, and all patches <0.5 as being *unfavourable* for the species.

### Distribution of suitable habitat

#### Catchments where purple-crowned fairy-wrens occur

**Figure 2:**
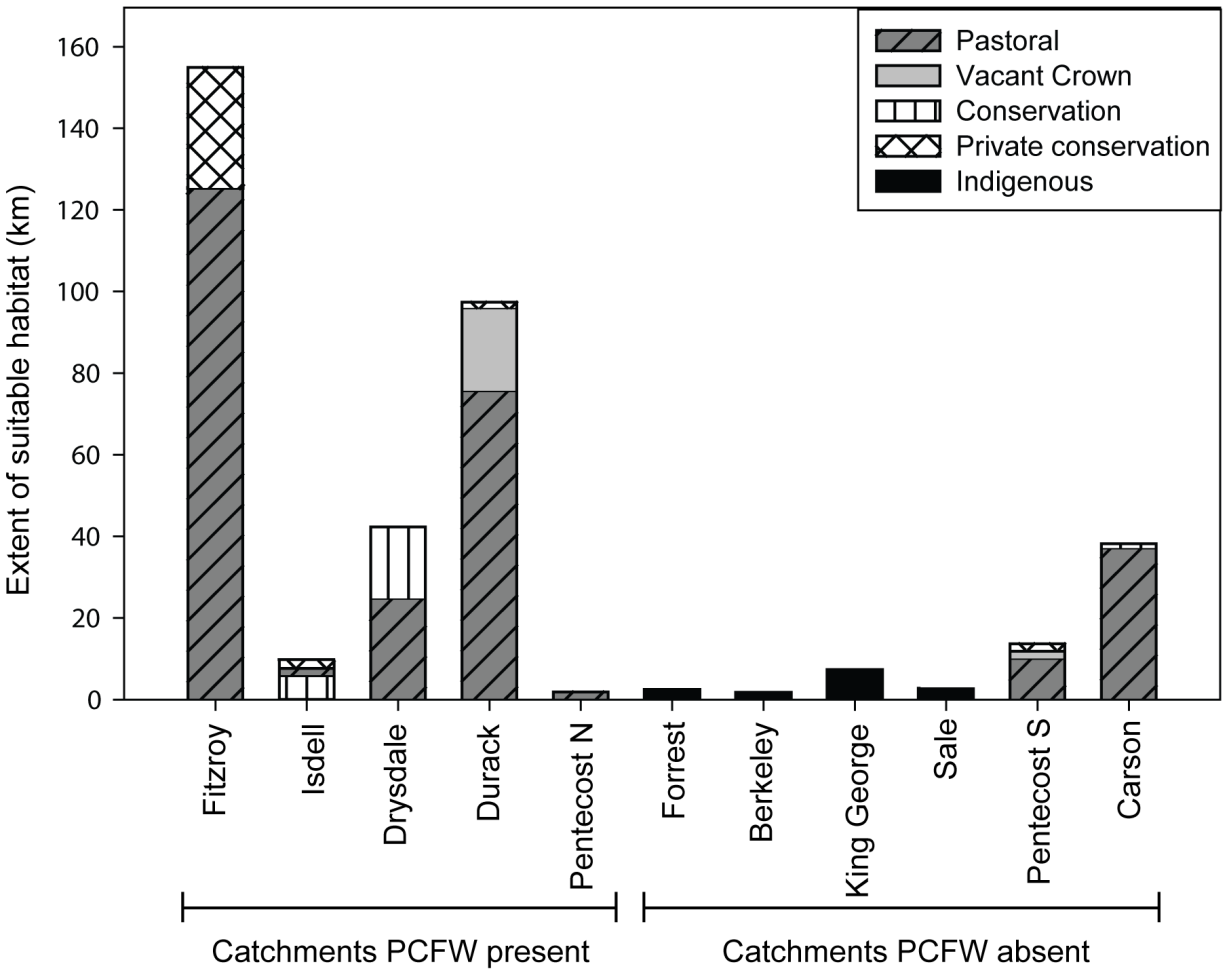
The predicted extent of suitable habitat for the purple-crowned fairy-wren with land tenure. Catchments are designated PCFW present if they belong to the current distribution of the purple-crowned fairy-wren, and PCFW absent if the species does not occur on them. The Ord and Charnley Rivers, although surveyed, are not depicted as no suitable habitat was detected on these rivers.

**Figure 3:**
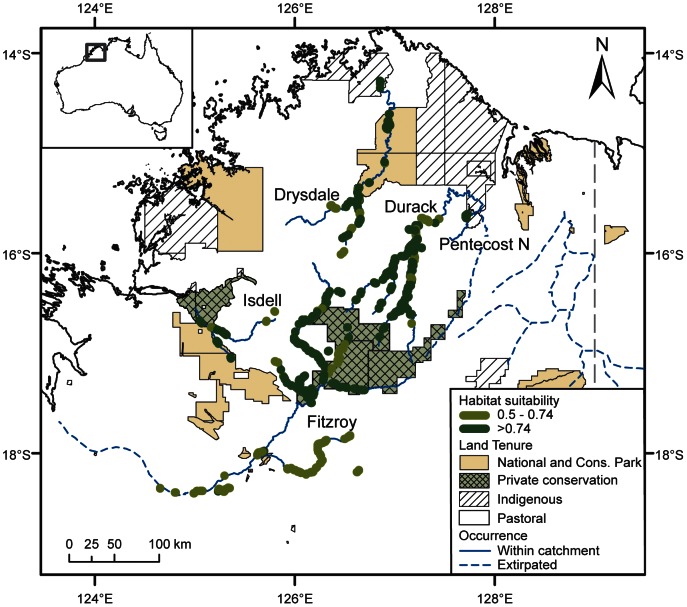
The predicted location of suitable habitat for the purple-crowned fairy-wren within the Kimberley region. Only the rivers where the species now occurs, or known to have previously occurred but has since disappeared, are indicated.

Of the catchments where the purple-crowned fairy-wren occurs, the Fitzroy, Durack and Drysdale contained large extents of suitable habitat, whereas the Isdell and northern Pentecost catchments contained a limited amount of suitable habitat (Figures 2 & 3). The vast majority of suitable habitat (77%) was located on pastoral lands, with only 17% located on conservation lands (private and government), and a small extent on vacant Crown Land (6%). No indigenous lands were present within the watersheds containing populations of purple-crowned fairy-wrens (Figure 3). Most habitat occurring on conservation land was located in three reserves: Mornington Wildlife Sanctuary (private conservation; Fitzroy catchment), Drysdale National Park (conservation; Drysdale catchment), and King Leopold Conservation Park (conservation; Isdell catchment).

In the Fitzroy catchment, 125 km (81%) of suitable habitat was found on pastoral land and 29 km (19%) on conservation land (mostly on Mornington Wildlife Sanctuary, with a small extent in Geike Gorge National Park) (Figure 2). The majority of suitable habitat was located in the mid to upper sections of the catchment, on the Hann, Fitzroy, Adcock, and Throssell Rivers, as well as Annie Creek. Areas to the south of Dimond Gorge contained habitat that was generally unfavourable, with the exception of a few stretches of high quality habitat on the Margaret and Leopold Rivers (Figure 2).

The Durack catchment contained the second highest cumulative length of suitable habitat (Figure 2). The majority of habitat (76 km; 77%) was located on Karunjie (indigenous pastoral lease) and Wood River (vacant Crown Land; 20 km, 21%), with a small extent (1.4 km, 1%) on Marion Downs Wildlife Sanctuary (private conservation). The third highest length of suitable habitat was identified on the Drysdale catchment (Figure 2), where most habitat occurred on pastoral land (24.5 km, 58%), with the remainder (17.5 km, 42%) in Drysdale National Park (Figure 3).

The Isdell and northern sections of the Pentecost catchment contained comparably little habitat (Figure 2). Suitable habitat within the Isdell catchment was located on Bell Creek in the King Leopold Conservation Park (5.4 km), on a section of the Isdell River which forms a boundary between the eastern edge of the Conservation Park and the Artesian Range Wildlife Sanctuary (1.8 km) and on pastoral land (2 km). Within the area that was mapped on the northern Pentecost catchment, the population of purple-crowned fairy-wrens appears to be limited to five patches of suitable habitat (totalling 3 km), all on pastoral land (Figure 2 & 3).

#### Catchments where purple-crowned fairy-wrens do not occur

Suitable habitat was generally limited on catchments and waterways where purple-crowned fairy-wrens do not occur (Figure 2 & 3). For catchments that have never been known to contain populations of purple-crowned fairy-wrens, no suitable habitat was identified on the Charnley River, less than 5 km of suitable habitat was identified on each of the Forrest, Berkeley, Sale, and Calder, slightly more on the King George (7.3 km), and a fairly extensive amount was identified on the Carson in the northern Kimberley (38 km). Some habitat (11.8 km) was identified on the southern Pentecost catchment where sightings were previously reported [39]. No suitable habitat was mapped on the Ord catchment (Table 2) where the species was regularly reported until 2004 and now presumed extirpated [25].

### Population estimates

We estimate that between 1013 and 1524 territories of purple-crowned fairy-wrens equating to 2834 to 4878 individuals may be supported by the extent of suitable habitat available to this species in the Kimberley region (Table 6). Large populations were located on the Fitzroy, Durack and Drysdale, and two smaller populations were located on the Isdell and northern Pentecost catchments (Table 6). Many of the catchments where the species does not occur contain habitat that could only support very small, and perhaps unsustainable, populations. We estimate that only the Carson could potentially support a large population (355–600 individuals; Table 6).

**Table 6:** Estimates of the extent of suitable habitat and the resulting *theoretical* number of purple-crowned fairy-wrens, and their territories, that could occur on 14 catchments surveyed within the Kimberley region.

	Suitable habitat		*Territories*		*N*	
Catchment	Patches	Extent (km)	Lower	Upper	Lower	Upper
*PCFW present*						
Fitzroy	155	154	513	771	1434	2467
Isdell	21	9	30	46	85	147
Drysdale	50	42	140	212	393	677
Durack	111	97	324	486	905	1557
Pentecost N	5	3	6	9	17	30
Sub total	342	305	1013	1524	2834	4878
*PCFW absent*						
Ord	0	0	0	0	0	0
Forrest	5	2	8	12	22	38
Berkeley	5	2	6	9	16	27
King George	14	7	24	37	67	115
Sale	4	3	9	14	25	43
Calder	10	3	9	14	26	45
Charnley	0	0	0	0	0	0
Pentecost S	21	12	39	59	109	188
Carson	53	38	126	191	355	611
Total	454	372	1234	1860	3455	5947

Population size estimates were based on the predicted extent of suitable habitat within each catchment and information on group size and territory length. Present = status of purple-crowned fairy-wren distribution within the catchment; suitable habitat >0.5 HS; *N* = absolute population size. Lower and upper estimates are based on 95% confidence intervals of average length of territories and mean number of birds per territory.

## Discussion

Our survey shows that habitat suitable for the purple-crowned fairy-wren was limited in extent and had an extremely patchy distribution along waterways surveyed within the Kimberley region. The five sub-populations of purple-crowned fairy-wrens occurring in the Kimberley region were restricted to 305 km of potentially suitable habitat that was dispersed as 342 patches along 2700 km of waterway (11% of the waterways; Table 6). Sub-populations spanned multiple patches of habitat on each of the five catchments where the species occurs, and the viability of each of the five sub-populations was likely to vary depending on population size and connectivity [40], [41]. Populations most at risk of extinction were the extremely small Isdell and northern Pentecost sub-populations, while the larger Fitzroy, Durack and Drysdale sub-populations were more secure.

### Determinants of distribution

The distribution of the purple-crowned fairy-wren in the Kimberley region appears to be influenced by the extent of suitable habitat as well as barriers to dispersal. The species occurred as three large populations (Fitzroy, Durack and Drysdale) and two smaller populations (Isdell and northern Pentecost) on catchments that are clustered in the central Kimberley. Habitat was insufficient to support populations of fairy-wrens on the other catchments that were surveyed (Forrest, Berkley, King George, Sale, Calder, Charnley and Ord), except potentially for the Carson and the southern section of the Pentecost catchments (Figure 2). Although suitable habitat has been degraded within the Ord catchment and on some waterways where purple-crowned fairy-wrens occur [17], [19], habitat appears to be naturally limited on the northern coastal waterways (Forrest, Berkely, King George and Carson) which are recognized as some of the least stressed waterways in Australia [42].

In addition to the influence of habitat availability, the distribution of the purple-crowned fairy-wren is likely affected by its dispersal capabilities. Most purple-crowned fairy-wren dispersal occurs along waterways (Skroblin unpublished data). In high quality habitat average natal dispersal is less than 3 km of river distance, with very rare movements of up to 70 km of river distance (M. Hall unpublished data). Overland dispersal has not been observed and is suggested by population genetics to be uncommon (Skroblin unpublished data). It is doubtful therefore, that the species is capable of colonising remote waterways. For instance, colonisation of the Carson catchment, which contains a large extent of habitat (38 km), maybe impeded by the Carson Escarpment and the Ashton Range, which separate the Carson from the nearest population on the Drysdale River by an overland distance of greater than 30 km.

The absence of the purple-crowned fairy-wren from the southern Pentecost catchment may be influenced by both habitat availability and dispersal capabilities. It is unclear how habitat availability has changed on the Chamberlain River since large numbers of the species were reported in the early 20^th^ century [39]. However, the current absence of the species from the southern section of the catchment may be due to insufficient habitat (12 km of habitat along approximately 100 km of waterway) and/or an inability of the species to re-colonise through the 100 km long Chamberlain Gorge which is naturally devoid of riparian vegetation. Further research is required to investigate whether other factors, such as patterns of habitat fragmentation [8], [43], [44], may also limit occurrence of the species before assisted colonisation or re-colonisation of areas lacking purple-crowned fairy-wrens could be considered.

### Conservation approach – regional-scale

Habitat for the purple-crowned fairy-wren occurs mainly on pastoral land and was widely dispersed along waterways in the Kimberley region. A conventional system of reserves will therefore neither capture a large enough sub-population of wrens to ensure their persistence, nor safeguard connectivity between sub-populations and thus the maintenance of key population processes such as dispersal [45]. Moreover, the key threats to purple-crowned fairy-wrens and other biodiversity in the Kimberley (changes in fire patterns, introduced herbivores, feral cats, invasive weeds) currently affect all tenures in the region more or less indiscriminately, so that tenure designation as conservation land does not necessarily confer protection unless actively managed [46].

Conservation of the purple-crowned fairy-wren requires improved land management at a regional-scale to protect riparian habitat across all tenures. It is likely that the persistence of many other species in northern Australia, including those with cryptic population structure, will require landscape-scale conservation approaches [47], [48]. This will entail cooperation among multiple land holders to collaborate on stewardship for multiple goals, including production, ecological sustainability and biodiversity conservation [49], [50]. The positive outcomes of such an approach have already been demonstrated in the Kimberley region by two programs in particular. The regional donkey control program managed by the Department of Food and Agriculture Western Australia has reduced the standing herd of donkeys in the region from around 600,000 to less than 20,000 (M. Everrit, pers.comm.). Similarly, EcoFire [51], is a partnership between landholders, private conservation and government agencies to manage fire cooperatively over 4 million hectares of the central and north Kimberley. This project delivers a prescribed burning program that has reduced the incidence of extensive, intense fires. An associated monitoring program for selected biological indicators has demonstrated, amongst other metrics, that the control of fire at a focal monitoring site coincided with the expansion and thickening of (fire-sensitive) riparian vegetation and an increase in the population size of purple-crowned fairy-wrens [51].

### Management directives

#### Landscape scale

The main threats to the riparian habitat of purple-crowned fairy-wrens (introduced herbivores, frequent intense fire, weed invasion) [17], [19], [24], are ubiquitous across the savannahs and tenures of northern Australia [22], [52], [53]. Thus, management goals for improving the persistence of purple-crowned fairy-wrens and other riparian specialists are similar across land tenures. Stock access to riparian areas needs to be controlled and the incidence of intense fires needs to be reduced. These management initiatives can benefit pastoral productivity as well as biodiversity. Provisioning alternative water sources away from waterways distributes grazing more uniformly and increases pasture utilisation [54] while concurrently reducing the impact of grazing and trampling on riparian vegetation structure [55]–[57], channel morphology and water quality [58]. Reduced incidence of intense fires will increase the availability of unburnt pasture for cattle (Letnic 2004) and decrease the economic losses pastoralist incur if it reduces the need for supplementary feeding and repairs to damaged infrastructure [59], [60].

#### Subpopulation scale

While conservation management of the purple-crowned fairy-wren is best undertaken at a landscape scale, conservation outcomes can be improved by directing specific actions at the sub-population scale. The most urgent conservation attention may be required by the small and isolated northern Pentecost and Isdell sub-populations. Both these sub-populations are genetically divergent and functionally isolated from other sub-populations (Skroblin unpublished data), and therefore at heightened risk of extinction due to their size [40].

The remnant habitat on the northern Pentecost catchment may support only 30 purple-crowned fairy-wrens (Table 6); a population size that is unlikely to be viable. Although the habitat on the Isdell may support a population that is several times larger than that on the Pentecost (Table 6), it was also largely restricted to one short section of waterway and thus at high risk of total degradation through single fire or flood events [41]. The northern Pentecost population occurs on pastoral land and could be protected by fencing to exclude grazing and fine-scale managed burning around habitat patches. The section of the Isdell population that occurs within King Leopold Conservation Park and may be best protected by heavily reducing the number of feral cattle and through careful, fine-scale fire management to limit the risk of extensive, intense fires affecting the riparian vegetation. The outcome of these fine-scale conservation actions would be enhanced by undertaking detailed on-ground surveys to assess the location of territories, the quality of habitat and fine-scale variation in threatening processes.

Although the other three populations on the Fitzroy, Durack and Drysdale are larger and thus at lower risk of immediate extinction, they are nevertheless threatened by continuing habitat degradation. These populations occur across many habitat patches on multiple properties and will thus benefit most from landscape-scale approaches to reducing threatening processes. Controlling access of stock to waterways and landscape management of fire should allow any degraded riparian habitat on these catchments to regenerate [61], and will help maintain the patches of high quality habitat that occur there. Securing the high density populations in the northern Fitzroy (on the Adcock, Hann, Throssell, Annie, and tributaries) may be of higher benefit than investment in populations in the southern part of the catchment where habitat was more highly fragmented (Figure 3) and degraded [19], and populations have already undergone significant decline. Once high density populations are considered secure, conservation efforts could consider rehabilitation and re-colonisation of areas where the species has become extinct e.g. lower Fitzroy and Ord catchment.

## Conclusion

This study presents a rare regional assessment of the size and distribution of a threatened species. It clearly indicates that landscape-scale conservation effort, across multiple tenures, is critical to preserving the widely dispersed and patchily distributed purple-crowned fairy-wren within the Kimberley region. The greatest benefit may be achieved by a combination of broad-scale actions to reduce the impact of threatening processes across sub-populations, and fine-scale targeted effort in areas where populations are most vulnerable. To be successful, such off-reserve approaches would require collaboration among multiple landholders with foreseeable benefits to both biodiversity and pastoral production. Such a landscape-wide collaborative approach to conservation may be vital for the protection of other species that are patchily distributed (both naturally and anthropogenically).

## Supporting Information

Table S1 Sections of waterways surveyed during aerial vegetation mapping in the Kimberley region. Latitude and Longitude is in decimal degrees. PCFW refers to whether the purple-crowned fairy-wren was detected.
(DOCX)

## Acknowledgments

We thank three anonymous reviewers for their insightful comments, as well as Andrew Cockburn and Michelle Hall for helpful advice on earlier drafts. We thank many volunteers for their enthusiasm, and Terry Webb for GIS support. The study was based out of the WildlifeLink Centre for Research and Conservation at Mornington Wildlife Sanctuary in the central Kimberley, which is owned and managed for conservation by the Australian Wildlife Conservancy.
