# Supplementary material for: Conservation of the Patchily Distributed and Declining Purple-Crowned Fairy-Wren (Malurus coronatus coronatus) across a Vast Landscape: The Need for a Collaborative Landscape-Scale Approach
Source: PLoS One. 2013 May 29;8(5):e64942. doi: 10.1371/journal.pone.0064942 (PMC3667184; doi:10.1371/journal.pone.0064942)
Supplement: Table S1 — Sections of waterways surveyed during aerial vegetation mapping in the Kimberley region. Latitude and Longitude is in decimal degrees. PCFW refers to whether the purple-crowned fairy-wren was detected. (DOCX) [file pone.0064942.s001.docx]

Table S1.

|  |  |  | Survey start | | Survey finish | |  |
| --- | --- | --- | --- | --- | --- | --- | --- |
| River system | No. | Name | Lat (° S) | Long (° E) | Lat (° S) | Long (° E) | PCFW |
| Fitzroy | 1 | Adcock River | -17.553 | 126.143 | -17.150 | 125.728 | y |
|  | 2 | Annie Creek | -17.495 | 126.113 | -17.534 | 126.110 | y |
|  | 3 | Bella Creek | -16.626 | 126.481 | -16.512 | 126.403 | y |
|  | 4 | Fitzroy River (south) | -18.087 | 124.180 | -18.193 | 124.491 | n |
|  | 5 | Fitzroy River (upper) | -17.499 | 126.684 | -18.393 | 124.575 | y |
|  | 6 | Grey Mare Creek | -17.363 | 126.104 | -17.368 | 126.094 | y |
|  | 7 | Hann River | -16.513 | 126.356 | -17.435 | 126.301 | y |
|  | 8 | Little Gold River | -18.000 | 126.491 | -18.001 | 126.766 | y |
|  | 9 | Manning Creek | -16.466 | 125.888 | -16.851 | 126.059 | y |
|  | 10 | Margaret River | -18.555 | 126.632 | -18.154 | 125.762 | y |
|  | 11 | O'Donnell River | -18.349 | 126.603 | -18.249 | 126.823 | n |
|  | 12 | Roy Creek | -17.465 | 126.183 | -17.539 | 126.197 | y |
|  | 13 | Spider Creek | -17.617 | 126.151 | -17.634 | 126.177 | y |
|  | 14 | Throssell River | -17.368 | 126.094 | -17.493 | 126.052 | y |
|  | 15 | Traine River | -16.919 | 126.546 | -17.332 | 126.305 | y |
|  | 16 | Clean Skin Creek | -17.153 | 126.452 | -17.055 | 126.764 | n |
|  | 17 | Yellow Creek | -17.079 | 126.404 | -17.138 | 126.303 | y |
|  | 18 | Traine River Trib | -16.917 | 126.545 | -16.759 | 126.505 | y |
| Isdell | 19 | Bell Creek North | -16.875 | 125.158 | -17.061 | 125.254 | y |
|  | 20 | Isdell River East | -16.720 | 125.933 | -16.571 | 125.807 | y |
|  | 21 | Isdell River West | -17.017 | 125.404 | -16.433 | 124.836 | y |
| Drysdale | 22 | Banjo Creek | -15.263 | 126.911 | -15.409 | 127.172 | y |
|  | 23 | Drysdale River | -15.984 | 125.984 | -14.247 | 126.937 | y |
|  | 24 | Gibb River | -15.620 | 126.625 | -16.252 | 126.366 | y |
| Durack | 25 | Blackfellow Creek | -16.300 | 126.978 | -16.756 | 126.756 | y |
|  | 26 | Chapman River | -16.701 | 126.545 | -16.044 | 127.201 | y |
|  | 27 | Durack River | -15.598 | 127.832 | -17.119 | 127.138 | y |
|  | 28 | Ellenbrae Creek | -15.845 | 126.753 | -15.899 | 127.220 | n |
|  | 29 | Leopold River | -17.766 | 126.646 | -18.304 | 126.647 | y |
|  | 30 | Wood River | -16.619 | 127.101 | -17.053 | 126.835 | y |
| Pentecost - north | 31 | Bindoola Creek | -15.703 | 127.853 | -15.784 | 127.723 | y |
|  | 32 | Pentecost River | -15.972 | 127.928 | -15.703 | 127.853 | n |
| Forrest | 33 | Forrest River | -15.201 | 127.810 | -15.226 | 127.297 | n |
| Berkley | 34 | Berkley River | -15.177 | 127.247 | -14.835 | 127.649 | n |
| King George | 35 | King George River | -14.544 | 127.315 | -14.068 | 127.311 | n |
| Sale | 36 | Sale River | -16.067 | 124.822 | -15.927 | 125.117 | n |
| Calder | 37 | Calder River | -16.063 | 125.211 | -16.369 | 124.953 | n |
| Charnley | 38 | Charnley River | -16.259 | 125.363 | -16.303 | 125.894 | n |
| Ord | 39 | Bow River | -16.827 | 128.364 | -16.696 | 128.644 | n |
|  | 40 | Negri | -17.090 | 128.889 | -17.073 | 129.000 | n |
|  | 41 | Ord River | -16.617 | 128.640 | -17.213 | 128.884 | n |
|  | 42 | Turkey Creek | -17.016 | 128.226 | -16.827 | 128.364 | n |
| Pentecost - south | 43 | Chamberlain River | -17.236 | 127.063 | -15.972 | 127.928 | n |
|  | 44 | Chamberlain Trib | -17.280 | 127.242 | -17.324 | 127.261 | n |
| Carson | 45 | Carson River | -14.425 | 126.639 | -15.220 | 126.514 | n |
|  | 46 | King Edward River | -14.967 | 126.128 | -14.425 | 126.639 | n |
|  | 47 | Morgan River | -14.753 | 126.777 | -14.974 | 126.314 | n |
